# Clinical Associations of Thyroid Hormone Levels with the Risk of Atherosclerosis in Euthyroid Type 2 Diabetic Patients in Central China

**DOI:** 10.1155/2020/2172781

**Published:** 2020-07-03

**Authors:** Limin Wang, Tingting Chen, Jiawei Yu, Huijuan Yuan, Xinru Deng, Zhigang Zhao

**Affiliations:** ^1^Department of Endocrinology, People's Hospital of Zhengzhou University, Henan Provincial People's Hospital, Zhengzhou, Henan 450003, China; ^2^School of Food Science, State Key Laboratory of Food Science and Technology, Nanchang University, Nanchang, Jiangxi 330047, China; ^3^Department of Nephrology, The People's Liberation Army No. 988 Hospital, Zhengzhou, Henan 450003, China

## Abstract

**Background:**

Thyroid function is associated with the etiology and pathogenesis of type 2 diabetes (T2D) and potentially contributes to the development of the complications of T2D. The association of thyroid hormones with atherosclerosis in euthyroid T2D patients is not clear.

**Purpose:**

To investigate the association of thyroid hormone levels with the risk of developing atherosclerosis in euthyroid T2D patients in Central China.

**Methods:**

This cross-sectional study recruited 910 euthyroid T2D patients from Henan Provincial People's Hospital, China. Association among hemoglobin A1c (HbA1c), thyroid hormones, and the prevalence of atherosclerosis was assessed by multivariable Cox models after adjusting for covariates including age, BMI, duration of T2D, smoking status, SBP, TC, family history of T2D, and medications on hyperlipidemia.

**Results:**

Among all 910 subjects, 373 were diagnosed with atherosclerosis. There were 523 females and 387 males included in this study. The mean age was 51.9 years. The average BMI was 25.3 kg/m^2^. Low-normal serum-free triiodothyronine (FT3) levels (3.50–4.17 pmol/L) were associated with a high prevalence of atherosclerosis. Comparing with low-normal FT3, the prevalence ratio in patients with mid- (4.17–4.83 pmol/L) and high-normal FT3 level (4.83–6.50 pmol/L) is 0.74 (95% CI 0.56 to 0.97, *p*=0.029) and 0.63 (95% CI 0.46 to 0.87, *p*=0.005) after adjusting for covariates. High level of free thyroxine (FT4) also had decreased risk for atherosclerosis. Thyroid-stimulating hormone (TSH) and FT3 to FT4 ratio did not show significant association with the development of atherosclerosis.

**Conclusion:**

T2D patients with low but clinically normal FT3 level are more likely to develop macrovascular complications comparing with those with mid- and high-normal FT3 level.

## 1. Introduction

Type 2 diabetes (T2D) is a metabolic disease characterized by increased blood sugar levels and insulin resistance. T2D is also an endocrine disease associated with the dysfunction of endocrine hormones, especially thyroid hormones [1, 2]. Epidemiological investigations have found that the prevalence of thyroid dysfunction in diabetic populations is significantly higher than that in nondiabetic people [3, 4]. In turn, the thyroid dysfunction adversely affected the carbohydrate metabolism, thereby accelerating the pathogenesis of T2D and the development of the diabetic complications.

Atherosclerotic disease is the major complication of type 2 diabetes (T2D) and the leading cause of death in T2D. The development of atherosclerosis is associated with increased duration of T2D. Measures of atherosclerotic burden are associated with clinically manifest cardiovascular diseases (CVD) in subjects with T2D. It is believed that obesity and insulin resistance contributed to the association between T2D and CVD [5]. Hemoglobin A1c (HbA1c) is an important biomarker for long-term glucose homeostasis in T2D patients. van 't Riet [6] reported that HbA1c is an independent predictor of nonfatal CVD in nondiabetic individuals. HbA1c higher than 8.5% was found to predict excess risk of all‐cause CVD [7]. And the variability of HbA1c levels in a prospective study predicted the development of renal and cardiovascular complications [8].

Thyroid dysfunction and subclinical dysfunction were also associated with T2D and its complications [9]. However, the influence of thyroid hormones on CVD was inconclusive. Pérez et al. [10] found that thyrotropin (TSH) and free thyroxine (FT4) concentrations do not affect cardiovascular disease and mortality in euthyroid peritoneal dialysis patients. Others found that subclinical hypothyroidism is a risk factor for CVD in T2D patients [11]. Treatment on thyroid dysfunction in subclinical hypothyroidism T2D patients reduced the risk of CVD [12]. FT4 levels in middle-aged and elderly subjects were positively associated with atherosclerosis, independent of cardiovascular risk factors [13]. However, not many studies have evaluated the thyroid parameters and atherosclerosis in euthyroid T2D patients. Whether thyroid status is an independent factor predicting atherosclerosis in euthyroid T2D patients is still unknown. In addition, high HbA1c level was found in hypothyroid patients. Treatment on thyroid function normalized HbA1c level without affecting blood glucose level [14]. Therefore, this cross-sectional study explored the association with HbA1c levels and thyroid functions and their association with the prevalence of atherosclerosis in euthyroid T2D patients. The result of this study will provide a reliable theoretical basis for seeking more precise treatment pathways and prognosis in clinical work.

## 2. Materials and Methods

### 2.1. Design, Settings, and Study Population

This study recruited inpatients who visited the Department of Endocrinology in Henan Provincial People's Hospital from January 2008 to January 2017. Participants who were diagnosed as T2D according to 1999 World Health Organization (WHO) criteria and have normal thyroid function were recruited. T2D was defined as (1) a causal plasma glucose ≥11.1 mmol/L any time of the day and the classic symptoms of hyperglycemia including polyuria, polydipsia, and unexplained weight loss; (2) or fasting plasma glucose levels ≥7.0 mmol/L or 2 h blood glucose level ≥11.1 mmol/L during oral glucose tolerance test (OGTT).

Normal thyroid function is defined as thyroid hormones in the normal range which was included in this study. The normal reference range of the thyroid hormones is TSH (0.55–4.78 *μ*IU/mL), serum-free triiodothyronine (FT3) (3.5–6.5 pmol/L), and FT4 (11.5–22.7 pmol/L). The cut-off values for the tertile distribution of FT3 were 4.21 and 4.63 pmol/L, FT4 were 15.2 and 17.2 pmol/L, TSH were 1.63 and 2.69 *μ*IU/mL, and ratio of FT3 to FT4 were 0.26 and 0.29.

Macrovascular complications were defined as atherosclerosis of the aorta, coronary, basilar, carotid, and renal arteries based on diagnoses of echocardiography and vascular ultrasonography. The exclusion criteria followed a previous study [4]. Specifically, those with type 1 diabetes, latent immune diabetes of the adults, gestational diabetes, and other types of diabetes, pregnancy, neoplasms, thyroid diseases, including overt and subclinical hyper/hypothyroidism, and a previous history of endocrine diseases including thyroid disease, pituitary, and hypothalamic diseases, as well as any major medical condition in 6 months preceding the study (i.e., liver, kidney, and heart failure and any type of cancer), were excluded from this study. Participants who took medications including thyroid supplementation and antithyroid agents, such as IFNc, amiodarone, lithium, and corticosteroids, were excluded. Other medications that affected the thyroid hormone levels, including glucocorticoids, estrogens, antipsychotics, lithium, immunomodulators, sedatives, histamine drugs, and iodine-containing drugs, were also excluded. The present study was approved by the Institutional Ethics Committee of Henan Provincial People's Hospital (no. 2018-lunshen-17). Informed consent was obtained from all participants.

### 2.2. Clinical and Laboratory Parameters

Participants recruited in this study received a general physical examination. Body weight and height were measured for body mass index (BMI) calculation (kg/m^2^, weight divided by squared height). Systolic blood pressure (SBP) and diastolic blood pressure (DBP) were measured twice consecutively, and the average was calculated. Blood samples of subjects were drawn after overnight fasting. HbAlc was determined by high-performance liquid chromatography (Hemoglobin Analyzer D-10, Bio-Rad Laboratories, Berkeley, CA, USA). Serum-free triiodothyronine (FT3), free thyroxine (FT4), and thyroid-stimulating hormone (TSH) were determined by chemiluminescence tests (Siemens ADVIA Centaur XP). Other biochemical indicators including fasting blood glucose (FBG), total cholesterol (TC), triglyceride (TG), high-density lipoprotein cholesterol (HDL-C), and low-density lipoprotein cholesterol (LDL-C) were analyzed by a biochemistry analyzer (Roche Diagnostics). A screening questionnaire regarding the age, gender, smoking status, family history of diabetes, and detailed medical history was filled by each patient.

### 2.3. Variable Explanation

The exposure variables in this study are tertile of thyroid hormones and HbA1c levels. Subjects who were recruited in this study were divided into three groups according to the tertile of FT3, FT4, FT3 to FT4 ratio (FT3/FT4), TSH, and HbA1c levels. Confounding variables were adjusted in the analysis after test for collinearity, including age, sex, BMI, smoking status, blood pressures (SBP and DBP), and lipid profiles (TC, TG, HDL-C, and LDL-C). The primary outcome in this study was the prevalence of atherosclerosis of the coronary arteries, peripheral arteries, and cerebrovascular based on diagnoses of echocardiography and vascular ultrasonography. The diagnose criteria include changes in the blood vessel lumen with buildup of plaque and/or lumen occlusion; narrowing lumen and no blood flow signal; and thickening of the blood vessel wall, including the common carotid artery 2-3 cm below the bifurcation was more than 1.2 mm thicker than intima-media for the carotid artery, and lower extremity blood vessel wall was more than 1.0.

### 2.4. Statistical Analysis

All statistical analyses were performed using the R statistical language (v.3.4.3). The normality test was conducted on all variables obtained. Continuous variables with the normal distribution were summarized as mean (standard deviation, SD), and nonnormal-distributed variables were reported as median (25% quantile, 75% quantile). The category variables were reported as count (percentage). Student's *t*-test and chi-square test/Fisher's exact test were used to determine the homogeneity of baseline characteristics. For data normally distributed, independent *t*-test and ANOVA test were used. And for data that were not normal, nonparametric tests, including Mann–Whitney test and Kruskal–Wallis test, were used instead. For count data, chi-square test was used.

To investigate the relationship between thyroid function and HbA1c levels, quantile regression was conducted with adjusting for BMI, duration of T2D, smoking status, TG, and history of hyperlipidemia. Then, the prevalence of atherosclerosis was used as the dependent variable, and tertile of HbA1c, FT3, FT4, and TSH concentrations were used as independent variables. Cox regression was used to assess the association between the tertile HbA1c and thyroid status with the prevalence of atherosclerosis after adjusting for covariates [15]. The risk period was set as a constant. The prevalence ratio was calculated based on the hazard rate ratio estimated by Cox regression. The confidence interval was adjusted with robust variance. To select the covariates, literature studies and the collinearity test on covariates were considered. Model 1 was adjusted for age and BMI. Model 2 was adjusted for age, BMI, duration of T2D, smoking status, SBP, TC, family history of T2D, and medications on hyperlipidemia. The statistical tests were two-sided, and a *p* value less than 0.05 was considered statistically significant. The significance level was marked as ^*∗*^ when *p* < 0.05, ^*∗∗*^ when *p* < 0.01, and ^*∗∗∗*^ when *p* < 0.001.

## 3. Results

### 3.1. Clinical Characteristics of the Study Population

**Figure 1:**
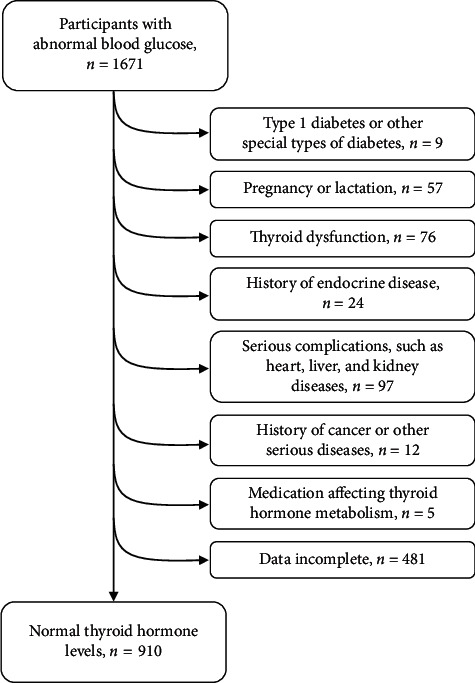
Flowchart of participant enrolment based on eligibility criteria.

We included a total of 910 participants with T2D and normal thyroid function based on the eligibility criteria (Figure 1). The baseline characteristics of the total population are shown in Table 1. The age (mean (SD)) of the included participants was 51.9 (13.1) years. The BMI was 25.3 (3.8) kg/m^2^. There were 523 females and 387 males included in this study. Among 910 patients, 373 were diagnosed with atherosclerosis. There was no significant difference on gender, HbA1c levels, and FBG. The age of T2D patients without atherosclerosis with an average of 46.3 (11.4) years was significantly younger comparing with that with atherosclerosis with an average of 59.9 (11.0) years. The duration of T2D in the nonatherosclerosis group was 3 [0.6, 6] years, significantly shorter than that of 10 [7, 15] years in the atherosclerosis group. The number of participants who had diseases including CVD, hypertension, and hyperlipidemia was all significantly higher in the atherosclerosis group. Thyroid hormones including FT3 and FT4 were significantly higher in people without atherosclerosis comparing with those with atherosclerosis. TSH levels were not significantly different between two groups. The lipid profiles showed no significant difference except TG. In other words, in addition to the common recognized risk factors, the prevalence of atherosclerosis is associated with FT3 and FT4 levels, but not with HbA1c levels.

### 3.2. Association of Thyroid Function with HbA1c

**Figure 2:**
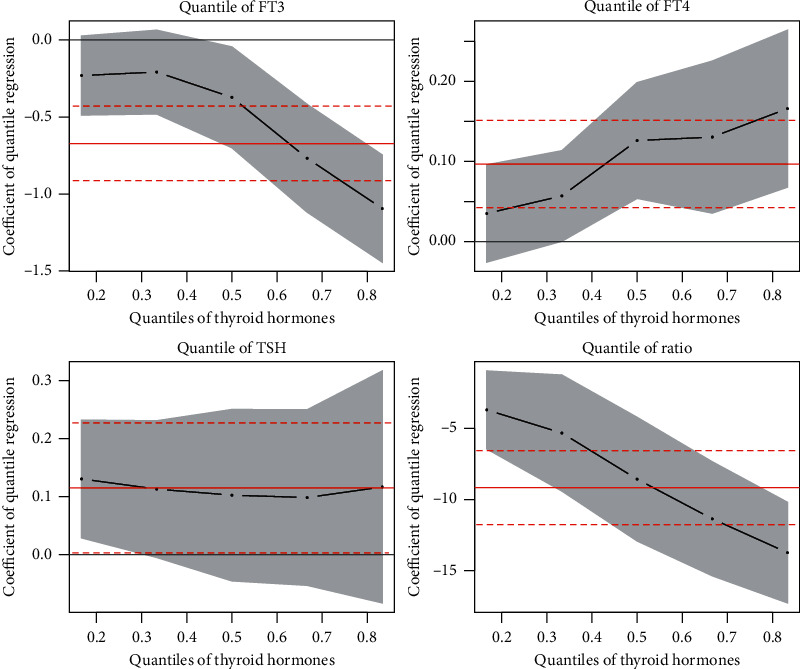
Coefficients of quantile regression between HbA1c levels and thyroid hormones after adjusting for BMI, duration of T2D, smoking status, TG, and history of hyperlipidemia.

In order to explore the association of HbA1c with the thyroid function, quantile regression between HbA1c levels and thyroid function was conducted with adjustment for BMI, duration of T2D, smoking status, TG, and history of hyperlipidemia. The coefficient of the quantile regression was plotted with 95% confidence interval (Figure 2). The coefficient between HbA1c and FT3 was −0.37 (−0.7, −0.01), −0.77 (−1.13, −0.42), and −1.09 (−1.5, −0.68) for quantiles 0.5, 0.667, and 0.833, indicating a significant negative correlation between HbA1c and FT3. With the increase of FT3 levels, the correlation coefficient between HbA1c and FT3 was further decreased. Opposite trend was observed between HbA1c and FT4. The coefficient was increased between HbA1c and FT4 with the increase of FT4, 0.03 (−0.05, 0.08), 0.06 (0, 0.15), 0.13 (0.02, 0.2), 0.13 (0.03, 0.25), and 0.17 (0.04, 0.3) for each quantile. The correlation between HbA1c and TSH: the confidence intervals of the coefficients between these two had zeros included, indicating no significant correlation, 0.13 (0.01, 0.28), 0.11 (−0.04, 0.28), 0.1 (−0.06, 0.32), 0.1 (−0.04, 0.25), and 0.12 (−0.17, 0.32) for each quantile. HbA1c showed a negative correlation with FT3/FT4 ratio. The correlation was stronger when the FT3/FT4 ratio was higher, −3.7 (−6.32, −0.08), −5.33 (−9.6, −0.97), −8.57 (−13.35, −3.91), −11.36 (−16.84, −6.75), and −13.74 (−17.16, −9.86) for each quantile.

### 3.3. Association of Thyroid Function and HbA1c with the Prevalence of Atherosclerosis

The correlations between HbA1c and the prevalence of atherosclerosis in T2D patients are revealed by previous studies. However, we found that HbA1c was not significantly correlated with the prevalence of atherosclerosis in this euthyroid T2D population. After dividing the participants into tertile based on HbA1c levels, the prevalence of atherosclerosis was not significantly correlated with HbA1c levels (Table 2).

Cox regression was also conducted between the prevalence of atherosclerosis and the thyroid hormones (Table 3). We have found a graded risk decrease in the prevalence of atherosclerosis with the increase in FT3 tertile. Model 1 was adjusted for age and BMI. In this model, mid- and high-normal FT3 levels showed smaller prevalence ratios 0.71 (95% CI 0.55 to 0.92, *p*=0.009) and 0.52 (95% CI 0.39 to 0.70, *p* < 0.001), respectively, comparing with the low-normal levels of FT3. In the model adjusted for duration of T2D, smoking status, systolic blood pressure, total cholesterol, family history of T2D, and medications on hyperlipidemia in addition to age and BMI, the prevalence ratio of mid-normal FT3 levels (PR 0.74 (95% CI 0.56 to 0.97), *p*=0.029) and high-normal FT3 levels (PR 0.63 (95% CI 0.46 to 0.87), *p*=0.005) was also smaller than low-normal FT3 levels. Similar trend of decreased risk was also found with FT4 tertile. Only high level of FT4 (15.2 to 17.2 pmol/L) was significantly lower than low level of FT4 (11.5 to 15.2 pmol/L) (PR 0.72 (95% CI 0.55 to 0.95), *p*=0.021). Comparing with the low-normal groups, the prevalence ratios of TSH levels and FT3/FT4 ratio in this euthyroid population were not significantly different for the prevalence of atherosclerosis.

## 4. Discussion

In this cross-sectional study, we investigated the association of HbA1c and thyroid hormones with the prevalence of atherosclerosis. We have found that low-normal FT3 levels are associated with increased risk of atherosclerosis after adjusting for the traditional known risks including age, duration of T2DM, smoking, BMI, SBP, TC, family history of DM, and lipid drugs. And HbA1c levels show no significant association with atherosclerosis using the same Cox regression. A weak correlation between HbA1c levels and thyroid parameters was found with FT3, FT4, and FR3/FT4 ratio, but not with TSH. This finding indicated that low-normal FT3 levels may be potential risk factors for atherosclerosis. Comparing to that, HbA1c levels did not have association with atherosclerosis in this study, even though HbA1c levels were correlated with FT3 levels.

As the leading cause of morbidity and mortality in T2D complications, CVD is often conjoined with T2D. Therefore, it is important to monitor the risk factors in T2D patients. Age, obesity, smoking status, alcohol use, and health conditions related to blood lipid and blood pressure are all risk factors for CVD and T2D. After adjusting for these factors, the risk of having CVD is still 4 times higher in T2D patients than people without T2D [16]. Besides CVD, other macrovascular conditions, including myocardial infarction, stroke, and peripheral artery disease, are more likely to occur and recur in T2D patients than people without diabetes [17]. The pathogenesis of atherosclerosis in T2D is multifactorial. People with diabetes often developed central obesity, dyslipidemia, and hypertension, which are collectively considered as metabolic syndrome. These conditions independently or cumulatively contribute to the risk of CVD in the long term. The major target in macrovascular complications in T2D is the vascular endothelium [17]. Also, the hyperinflammation that caused by diabetes damaged the endothelial and deeper layers. Hyperglycemia was also associated with increased intracellular acidosis, which might contribute to a transient ischemic attack or stroke.

In addition to insulin, endocrine hormones, especially thyroid hormone, also play an important role in the pathogenesis of diabetes. In this cross-sectional study, we have found a negative correlation between HbA1c and FT3 levels. However, only FT3 levels were negatively associated with the increased risk of atherosclerosis. HbA1c levels show no significant association with atherosclerosis. Epidemiological studies have found that the rate of thyroid dysfunction in diabetics is significantly higher than that in nondiabetics [18]. Not only that, the risk of T2D also increased in patients with thyroid disease [2]. Both overt and subclinical hypo- and hyperthyroid dysfunction were found associated with HbA1c level and T2D disease. HbA1c levels were significantly higher in the hyperthyroid group when compared with hypothyroid and euthyroid groups [19]. In this euthyroid T2D population, we found that HbA1c was negatively correlated with FT3 and positively correlated with FT4 and TSH. Thyroid hormones regulate many processes in carbohydrate metabolism, including the absorption of glucose, hepatic gluconeogenesis, and glycogenolysis. Besides, thyroid hormone also influences the secretion of insulin. Therefore, thyroid dysfunction and glucose intolerance often occur together. T3 is the primary, biologically active hormone involved in glucose homeostasis. The FT3/FT4 ratio is a marker indicating the conversion rate from T4 to T3. Both parameters were negatively correlated with HbA1c. T4 is produced by the thyroid gland, FT4 reflecting the function of the thyroid gland and less correlated with glucose metabolism [20].

Thyroid dysfunction was a risk factor for cardiovascular disease mediated by the effects of thyroid hormones on lipid metabolism and blood pressure. Recently, high-normal HbA1c and TSH levels have been found to be the co-risk factor for coronary heart disease [21]. In patients having TSH levels TSH ≥10 and < 0.10 mIU/L, the heart failure events increased. Among thyroid hormones, FT3 is cardioprotective. In euthyroid subjects, TSH is an independent predictor of cardiovascular disease [22]. FT4 is associated with differing metabolic markers [10]. Recently, a study in the general population has found that serum FT4 levels predict the risk of T2D and CVD [23]. Low triiodothyronine (T3) has been associated with inflammation, malnutrition, and mortality in dialysis [24].

Thyroid hormone plays an important role in the pathogenesis of diabetes. The influence of the thyroid hormone on the secretion of various endocrine hormones resulted in dyslipidemia and hypertension, both of which contributed to the complex etiology and pathogenesis in atherosclerosis in T2D. The possible mechanism between HbA1c and thyroid hormones with atherosclerosis could be related to the metabolism status and/or the inflammation status. In T2D, the metabolic state of the body is disordered, same as energy utilization. The iodine pump function in thyroid follicular cells is limited, resulting in decreased iodine uptake of the thyroid gland, affecting organic iodine. The process of the thyroid hormone, particularly FT3, is reduced. The chronic low-grade inflammatory state caused by T2D induced hyperfunction of the hypothalamus-pituitary-adrenocortical axis. As a result, thyrotropin-releasing hormone secretion was inhibited and TSH reactivity decreased, leading to decreased synthesis of FT3. Furthermore, the insufficient insulin secretion due to hyperglycemia caused reduced T4 to T3 conversion and increased conversion of T4 to 3,3′5′´-triiodothyronine (an inactive form of T3), both resulting in decreased levels of FT3. While T4 is produced only by the thyroid gland, T3 is converted from T4 in peripheral tissues. It could explain that FT3, but not FT4, was correlated with the atherosclerosis prevalence in this study. Unlike euthyroid T2D patients, FT3 levels were increased but not decreased in euthyroid and nondiabetic adults [25]. The possible reason could be that, in adults with insulin resistance, T3 was elevated to directly stimulate the uptake of glucose. And compensatory mechanism disappeared in patients with T2D [20, 26].

## 5. Conclusion

In this study, we found that T2D patients with low but clinically normal FT3 level are more likely to develop macrovascular complications comparing with those with mid- and high-normal FT3 level. The prevalence of atherosclerosis is not associated with HbA1c. Therefore, it is recommended to perform periodic screening for thyroid hormones in euthyroid diabetic patients for early diagnosis and prevention of atherosclerosis. Its early detection allows the implementation of individualized and aggressive intervention program to reduce cardiovascular risk factors. The main limitation of this study is the cross-sectional study, so the causal relationship between thyroid function-related indicator HbA1c and resulting atherosclerosis in euthyroid T2D patients cannot be inferred, and further follow-up studies and larger prospective studies are needed.

## Figures and Tables

**Table 1:** Anthropometric characteristics of the study population with and without previously diagnosed CVD.

	Total	Without atherosclerosis	With atherosclerosis	*p* value	
*N*	910	537 (59.0%)	373 (41.0%)		
Age (years)	51.9 (13.1)	46.3 (11.4)	59.9 (11.0)	<0.001	^*∗∗∗*^
BMI (kg/m^2^)	25.3 (3.8)	25.5 (4.0)	24.9 (3.4)	0.044	^*∗*^
Gender (%)				0.126	
F	523 (57.5%)	320 (35.2%)	203 (22.3%)		
M	387 (42.5%)	217 (23.8%)	170 (18.7%)		

Smoking status (%)				0.005	^*∗∗*^
Nonsmoker	529 (58.1%)	289 (31.8%)	240 (26.4%)		
Ex-smoker	110 (12.1%)	73 (8.0%)	37 (4.1%)		
Smoker	271 (29.8%)	175 (19.2%)	96 (10.5%)		

Years of T2D	5 [2, 10]	3 [0.6, 6]	10 [7, 15]	<0.001	^*∗∗∗*^
HbA1c (%)	8.4 [7.2, 9.9]	8.4 [7.1, 9.9]	8.4 [7.4, 9.9]	0.218	
FPG	8.4 [6.8, 10.7]	8.3 [6.8, 10.5]	8.7 [7.0, 10.9]	0.343	

Thyroid hormones					
FT3 (pmol/L)	4.41 [4.11, 4.76]	4.5 [4.19, 4.87]	4.26 [3.95, 4.6]	<0.001	^*∗∗∗*^
FT4 (pmol/L)	16.14 [14.64, 17.75]	16.38 [14.76, 18.23]	15.68 [14.42, 17.46]	0.001	^*∗∗*^
TSH (*μ*IU/mL)	2.11 [1.36, 3.10]	1.99 [1.30, 3.04]	2.21 [1.43, 3.17]	0.083	
FT3/FT4	0.27 [0.25, 0.31]	0.28 [0.25, 0.31]	0.27 [0.25, 0.3]	0.328	

Blood pressure					
SBP (mmHg)	135 [122, 149]	132 [120, 145]	138 [126, 153]	<0.001	^*∗∗∗*^
DBP (mmHg)	85 [80, 90]	86 [80, 90.75]	83 [79, 90]	0.013	^*∗*^

Lipid profiles					
TC (mmol/L)	4.88 [4.2, 5.62]	4.83 [4.20, 5.58]	4.92 [4.22, 5.67]	0.582	
TG (mmol/L)	1.62 [1.10, 2.63]	1.71 [1.11, 2.81]	1.55 [1.1, 2.27]	0.029	^*∗*^
HDL-C (mmol/L)	1.11 [0.95, 1.27]	1.1 [0.94, 1.26]	1.12 [0.96, 1.30]	0.252	
LDL-C (mmol/L)	2.92 [2.36, 3.5]	2.95 [2.35, 3.48]	2.92 [2.36, 3.53]	0.868	

T2D treatments					
Diet	107 (11.8%)	96 (10.5%)	11 (1.2%)	<0.001	^*∗∗∗*^
Oral drugs	529 (58.1%)	357 (39.2%)	172 (18.9%)	<0.001	^*∗∗∗*^
Insulin injection	94 (10.3%)	53 (5.8%)	41 (4.5%)	0.662	
Oral drugs and insulin	180 (19.8%)	31 (3.4%)	149 (16.4%)	<0.001	^*∗∗∗*^

History of other diseases					
CVD	396 (43.5%)	108 (11.9%)	288 (31.6%)	<0.001	^*∗∗∗*^
Hypertension	532 (58.5%)	247 (27.1%)	285 (31.3%)	<0.001	^*∗∗∗*^
Hyperlipidemia	392 (43.1%)	156 (17.1%)	236 (25.9%)	<0.001	^*∗∗∗*^
Family history of T2D	469 (51.5%)	299 (32.9%)	170 (18.7%)	0.006	^*∗∗*^

Medications					
Hypertension	408 (44.8%)	156 (17.1%)	252 (27.7%)	<0.001	^*∗∗∗*^
Hyperlipidemia	242 (26.6%)	65 (7.1%)	177 (19.5%)	<0.001	^*∗∗∗*^

Data are expressed as mean (SD), median (25% quantile, 75% quantile), or count (percentage) depending on the variable type. The *p* value was calculated by independent two-sample *t*-tests, Mann–Whitney U test, or chi-square test accordingly.^*∗*^When *p* < 0.05, ^*∗∗*^when *p* < 0.01, and ^*∗∗∗*^when *p* < 0.001. BMI: body mass index; FPG: fasting plasma glucose; SBP: systolic blood pressure; DBP: diastolic blood pressure; TC: total cholesterol; TG: triglycerides; LDL-C: low-density lipoprotein cholesterol; and HDL-C: high-density lipoprotein cholesterol.

**Table 2:** Prevalence ratio of atherosclerosis based on Cox regression on each tertile of HbA1c levels.

	Variable	Model 1			Model 2		
		PR	CI	*p* value	PR	CI	*p* value
HbA1c							
Q1	Q1 < 7.6	1.00	Ref		1.00	Ref	
Q2	7.6 ≤ Q2 < 9.4	1.10	0.82 to 1.40	0.575	1.20	0.89 to 1.60	0.223
Q3	Q3 ≤ 9.4	1.10	0.86 to 1.50	0.369	1.20	0.86 to 1.60	0.335

Model 1: adjusted for age and BMI. Model 2: adjusted for age, BMI, duration of T2D, smoking status, SBP, TC, family history of T2D, and medications on hyperlipidemia. PR: prevalence ratio. CI: 95% confidence interval of PR.

**Table 3:** Prevalence ratio of atherosclerosis based on Cox regression on each tertile of thyroid hormone levels.

	Variable	Model 1			Model 2		
		PR	CI	*p* value	PR	CI	*p* value
FT3							
Q1	Q1 < 4.21	1.00	Ref		1.00	Ref	
Q2	4.21 ≤ Q2 < 4.63	0.71	0.55 to 0.92	0.009^*∗∗*^	0.74	0.56 to 0.97	0.029^*∗*^
Q3	Q3 ≤ 4.63	0.52	0.39 to 0.70	<0.001^*∗∗∗*^	0.63	0.46 to 0.87	0.005^*∗∗*^

FT4							
Q1	Q1 < 15.2	1.00	Ref		1.00	Ref	
Q2	15.2 ≤ Q2 < 17.2	0.89	0.68 to 1.20	0.389	0.97	0.74 to 1.30	0.851
Q3	Q3 ≤ 17.2	0.72	0.55 to 0.95	0.021^*∗*^	0.82	0.62 to 1.10	0.195

TSH							
Q1	Q1 < 1.63	1.00	Ref		1.00	Ref	
Q2	1.63 ≤ Q2 < 2.69	1.20	0.91 to 1.60	0.193	1.20	0.91 to 1.70	0.174
Q3	Q3 ≤ 2.69	1.20	0.90 to 1.60	0.229	1.20	0.88 to 1.60	0.256

FT3/FT4							
Q1	Q1 < 0.26	1.00	Ref		1.00	Ref	
Q2	0.26 ≤ Q2 < 0.29	1.10	0.85 to 1.50	0.458	1.10	0.84 to 1.50	0.434
Q3	Q3 ≤ 0.29	1.00	0.78 to 1.40	0.834	1.00	0.77 to 1.40	0.794

Model 1: adjusted for age and BMI. Model 2: adjusted for age, BMI, duration of T2D, smoking status, SBP, TC, family history of T2D, and medications on hyperlipidemia. PR: prevalence ratio. CI: 95% confidence interval of PR.

## Data Availability

The data supporting the findings of this study are available within the article.

## References

[1] Biondi B., Kahaly G. J., Robertson R. P. (2019). Thyroid dysfunction and diabetes mellitus: two closely associated disorders. *Endocrine Reviews*.

[2] Chen R. H., Chen H. Y., Man K. M. (2019). Thyroid diseases increased the risk of type 2 diabetes mellitus: a nation-wide cohort study. *Medicine (Baltimore)*.

[3] Islam S., Yesmine S., Khan S. A., Alam N. H., Islam S. (2008). A comparative study of thyroid hormone levels in diabetic and non-diabetic patients. *The Southeast Asian Journal of Tropical Medicine and Public Health*.

[4] Zhu Y., Xu F., Shen J. (2019). Prevalence of thyroid dysfunction in older Chinese patients with type 2 diabetes-A multicenter cross-sectional observational study across China. *PLoS One*.

[5] Poznyak A., Grechko A. V., Poggio P., Myasoedova V. A., Alfieri V., Orekhov A. N. (2020). The diabetes mellitus-atherosclerosis connection: the role of lipid and glucose metabolism and chronic inflammation. *International Journal of Molecular Sciences*.

[6] van ’t Riet E., Rijkelijkhuizen J. M., Alssema M. (2012). HbA1c is an independent predictor of non-fatal cardiovascular disease in a Caucasian population without diabetes: a 10-year follow-up of the Hoorn Study. *European Journal of Preventive Cardiology*.

[7] Xu L., Chan W. M., Hui Y. F., Lam T. H. (2012). Association between HbA1c and cardiovascular disease mortality in older Hong Kong Chinese with diabetes. *Diabetic Medicine*.

[8] Luk A. O. Y., Ma R. C. W., Lau E. S. H. (2013). Risk association of HbA1cvariability with chronic kidney disease and cardiovascular disease in type 2 diabetes: prospective analysis of the Hong Kong diabetes registry. *Diabetes/Metabolism Research and Reviews*.

[9] Han C., He X., Xia X. (2015). Subclinical hypothyroidism and type 2 diabetes: a systematic review and meta-analysis. *PLoS One*.

[10] Pérez S. R., Díez J. J., Bajo M. A. (2013). Thyrotropin and free thyroxine concentrations do not affect cardiovascular disease and mortality in euthyroid peritoneal dialysis patients. *Peritoneal Dialysis International: Journal of the International Society for Peritoneal Dialysis*.

[11] Chen H.-S., Wu T.-E. J., Jap T.-S. (2007). Subclinical hypothyroidism is a risk factor for nephropathy and cardiovascular diseases in Type 2 diabetic patients. *Diabetic Medicine*.

[12] Seo C., Kim S., Lee M. (2018). Thyroid hormone replacement reduces the risk of cardiovascular diseases in diabetic nephropathy patients with subclinical hypothyroidism. *Endocrine Practice*.

[13] Bano A., Chaker L., Mattace-Raso F. U. S. (2017). Thyroid function and the risk of atherosclerotic cardiovascular morbidity and mortality. *Circulation Research*.

[14] Bhattacharjee R., Thukral A., Chakraborty P. (2017). Effects of thyroid status on glycated hemoglobin. *Indian Journal of Endocrinology and Metabolism*.

[15] Barros A. J., Hirakata V. N. (2003). Alternatives for logistic regression in cross-sectional studies: an empirical comparison of models that directly estimate the prevalence ratio. *BMC Medical Research Methodology*.

[16] Buyken A. E., von Eckardstein A., Schulte H., Cullen P., Assmann G. (2007). Type 2 diabetes mellitus and risk of coronary heart disease: results of the 10-year follow-up of the PROCAM study. *European Journal of Cardiovascular Prevention & Rehabilitation*.

[17] Cade W. T. (2008). Diabetes-related microvascular and macrovascular diseases in the physical therapy setting. *Physical Therapy*.

[18] Wang C. (2013). The relationship between type 2 diabetes mellitus and related thyroid diseases. *Journal of Diabetes Research*.

[19] Tam A., Tam H., Usluogullari C. (2015). The levels of HbA1c in patients with thyroid dysfunction. *Gaziantep Medical Journal*.

[20] Benites-Zapata V. A., Urrunaga-Pastor D., Torres-Mallma C., Prado-Bravo C., Guarnizo-Poma M., Lázaro-Alcántara H. (2017). Is free triiodothyronine important in the development of insulin resistance in healthy people?. *Diabetes & Metabolic Syndrome: Clinical Research & Reviews*.

[21] Li H., Cui Y., Zhu Y., Yan H., Xu W. (2017). Association of high normal HbA1c and TSH levels with the risk of CHD: a 10-year cohort study and SVM analysis. *Scientific Reports*.

[22] Gómez-Zamudio J. H., Mendoza-Zubieta V., Ferreira-Hermosillo A. (2016). High thyroid-stimulating hormone levels increase proinflammatory and cardiovascular markers in patients with extreme obesity. *Archives of Medical Research*.

[23] Wolffenbuttel B. H. R., Van V.-O. J. V., Van W. R. P (2018). Serum FT4 levels predict incidence of type 2 diabetes and cardiovascular disease in the general population. *Endocrine Abstracts*.

[24] Garduño-Garcia J. d. J., Alvirde-Garcia U., López-Carrasco G. (2010). TSH and free thyroxine concentrations are associated with differing metabolic markers in euthyroid subjects. *European Journal of Endocrinology*.

[25] Urrunaga-Pastor D., Guarnizo-Poma M., Moncada-Mapelli E. (2018). High free triiodothyronine and free-triiodothyronine-to-free-thyroxine ratio levels are associated with metabolic syndrome in a euthyroid population. *Diabetes & Metabolic Syndrome: Clinical Research & Reviews*.

[26] Farasat T., Cheema A. M., Khan M. N. (2012). Hyperinsulinemia and insulin resistance is associated with low T3/T4 ratio in pre diabetic euthyroid pakistani subjects. *Journal of Diabetes and Its Complications*.

